# ‘Function First—Be Active, Stay Independent’—promoting physical activity and physical function in people with long-term conditions by primary care: a protocol for a realist synthesis with embedded co-production and co-design

**DOI:** 10.1136/bmjopen-2019-035686

**Published:** 2020-02-09

**Authors:** Rebecca-Jane Law, Lynne Williams, Joseph Langley, Christopher Burton, Beth Hall, Julia Hiscock, Val Morrison, Andrew Lemmey, Rebecca Partridge, Candida Lovell-Smith, John Gallanders, Nefyn Williams

**Affiliations:** 1 North Wales Centre for Primary Care Research, School of Health Sciences, Bangor University, Wrexham, UK; 2 School of Health Sciences, Bangor University, Bangor, Gwynedd, UK; 3 Lab4Living, Sheffield Hallam University, Sheffield, South Yorkshire, UK; 4 School of Allied Health Professions, Canterbury Christ Church University, Canterbury, Kent, UK; 5 Library and Archives Service, Bangor University, Bangor, Gwynedd, UK; 6 School of Psychology, Bangor University, Bangor, Gwynedd, UK; 7 School of Sport, Health and Exercise Science, Bangor University, Bangor, Gwynedd, UK; 8 PPI Research Partner, UK; 9 Department of Health Services Research, University of Liverpool, Liverpool, UK

**Keywords:** primary care, physical activity, evidence synthesis, realist, co-design

## Abstract

**Introduction:**

People with long-term conditions typically have reduced physical functioning, are less physically active and therefore become less able to live independently and do the things they enjoy. However, assessment and promotion of physical function and physical activity is not part of routine management in primary care. This project aims to develop evidence-based recommendations about how primary care can best help people to become more physically active in order to maintain and improve their physical function, thus promoting independence.

**Methods and analysis:**

This study takes a realist synthesis approach, following RAMESES guidance, with embedded co-production and co-design. Stage 1 will develop initial programme theories about physical activity and physical function for people with long-term conditions, based on a review of the scientific and grey literature, and two multisector stakeholder workshops using LEGO® SERIOUS PLAY®. Stage 2 will involve focused literature searching, data extraction and synthesis to provide evidence to support or refute the initial programme theories. Searches for evidence will focus on physical activity interventions involving the assessment of physical function which are relevant to primary care. We will describe ‘what works’, ‘for whom’ and ‘in what circumstances’ and develop conjectured programme theories using context, mechanism and outcome configurations. Stage 3 will test and refine these theories through individual stakeholder interviews. The resulting theory-driven recommendations will feed into Stage 4 which will involve three sequential co-design stakeholder workshops in which practical ideas for service innovation in primary care will be developed.

**Ethics and dissemination:**

Healthcare and Medical Sciences Academic Ethics Committee (Reference 2018-16308) and NHS Wales Research Ethics Committee 5 approval (References 256 729 and 262726) have been obtained. A knowledge mobilisation event will address issues relevant to wider implementation of the intervention and study findings. Findings will be disseminated through peer-reviewed journal publications, conference presentations and formal and informal reports.

**PROSPERO registration number:**

CRD42018103027.

**Strengths and limitations of this study**

- A realist approach facilitates explanation of the complex nature of promoting physical activity and physical function as part of the management of long-term conditions in primary care, paying attention to the contextual factors that shape how interventions are implemented and generate impact.
- The use of ‘Collective Making’ activities, including LEGO® SERIOUS PLAY® for programme theory development and co-design, will enable creative stakeholder engagement and expression through model-building, use of metaphor and story-telling.
- Engagement and co-production with multisectoral stakeholders throughout the synthesis, and the addition of the co-design and knowledge mobilisation stages, will develop recommendations that are grounded in the real world and address practice and policy challenges.
- Realist synthesis is about what works in what contexts, so the review recommendations will need further consideration and modification for application in different contexts.

## Introduction

Three out of four older adults living in developed countries such as the UK have long-term conditions,^1^ and the prevalence rises with age.^2^ Treatment and care for people with long-term conditions is estimated to account for £7 in every £10 of total UK health and social care expenditure, which will increase further as the population ages.^3^ This increasing prevalence is one of the biggest challenges facing our health and social care systems.^4^

Major contributors to this challenge are the decline in physical function and physical activity characteristic to people with long-term conditions. ‘Physical function’ is an individual’s capacity to undertake physical tasks and is one of the most important factors for quality of life.^5–7^ A different but related concept is ‘physical activity’, which can be defined as ‘any bodily movement produced by skeletal muscles that results in energy expenditure’.^8^ Physical activity helps to prevent or delay functional decline and loss of independence.^9–12^ Moreover, adults who become physically active later in life have similar mortality rates to those of lifelong exercisers.^13^ Helping people to be more physically active also has benefits for mental health and mood.^14^ Thus, improvement in physical activity and physical function has promising potential for substantially reducing costs to health and social care services.^15 16^

Caring for people with long-term conditions is a core component of primary care^17 18^ and primary care is uniquely placed to empower individuals and communities.^19^ However, management of long-term conditions has focused on the diagnosis and categorisation of disease, and the management of important mediators such as blood pressure and glycaemic control in diabetes,^20^ rather than any concomitant decline in physical function. Placing more emphasis on functional limitations may promote more pro-active, ‘whole-person’ and preventive care approaches, benefiting the patient and targeting healthcare resources more effectively.^21 22^ Organisational interventions targeting patient-specific difficulties (eg, functional ability) appear more likely to be effective,^23^ especially when the intervention is more comprehensive and better integrated into routine care.^24^

Previous reviews have explored the effects of physical activity interventions in sedentary adults and those with long-term conditions in the primary care setting.^25–28^ Barriers and facilitators to physical activity and the effectiveness of different modes of delivery have been explored.^29–32^ NICE guidance has recommended brief physical activity advice as a way to prevent dementia, disability and frailty in later life.^33 34^ However, while the links between physical activity and physical function are evident and the benefits of physical activity are clear, the best way for primary care to help people with long-term conditions to increase physical activity and reduce functional decline is uncertain.

Optimising physical function and physical activity is likely to involve a complex intervention, given the range of potential influences (eg, personal, social, condition and treatment), and the range of resources that activate different responses in different people.^35^ A comprehensive understanding of an intervention, what it does and how it works, can facilitate meaningful application and improve sustainability.^36^ Therefore, it is important to understand the underlying theory and the critical components (or ‘active ingredients’) of an intervention, and a methodology that focuses on this complexity is required. A realist approach will provide a contextualised, explanatory account and understanding of ‘what is it about a programme (or intervention) that works (or does not work) for whom, and in what circumstances’.^37–39^

As well as the interrogation of relevant theory-rich literature, realist evidence syntheses are participatory in nature. They draw on the lived experiences of service users and professionals providing services to identify ‘nascent’ individual theories based on their experiences.^40^ To facilitate this, creative methods from the field of co-design will ensure that the views of all stakeholders are included and embedded within the process. The co-produced theory and ideas from these stakeholders will feed back into the literature searches, refining the search criteria, adding an interpretative frame to interrogate the literature, corroborating and refuting the evidence. The resulting theories will then feed into a co-design stage where they will be further refined and prioritised before being applied to generate recommendations for service innovation and implementation.

## Aims and objectives

1. To identify and produce a taxonomy of physical activity interventions that aim to reduce functional decline in people with long-term conditions managed in primary care.
2. To work with patients, health professionals and researchers to uncover the complexity associated with the range of physical activity interventions in primary care, and how they directly or indirectly affect the physical functioning of people with long-term conditions.
3. To identify the mechanisms through which interventions bring about functional improvements in people with long-term conditions, and the circumstances associated with how the interventions are organised and operate within different primary care contexts.
4. To understand the potential impacts of these interventions across primary care and other settings.
5. To co-produce an evidence-based, theory-driven explanatory account in the form of refined programme theory.
6. To develop a new intervention through a co-design process with patients, health professionals and researchers.

## Method and analysis

The established steps for a realist synthesis will be followed which include: clarifying the scope of the review, developing initial programme theory, evidence searching and appraisal, extracting data, synthesising evidence to test and refine the programme theory, drawing conclusions and recommendations.^37^

Programme theory is defined here as ‘the theory built into every programme (or intervention)’ that addresses the facilitation of physical activity within primary care^41^ and will be developed as ‘context, mechanism and outcome (CMO) propositions’. The ‘context’ in this study refers to the ’settings within which programmes (or interventions) are placed, or pre-existing factors outside the control of programme designers (eg, people’s motivation, organisational contexts or structures)’.^42^ Mechanisms are sensitive to context and defined as ‘how programmes (or interventions) change, or provide the resources for, people’s decision-making (eg, empowerment or confidence building)’.^41^ ‘Outcomes’ may have single or multiple effects^40^ and can be related to process (eg, a change in behaviour) or impact (whether an intervention worked or not).^43^

**Figure 1:**
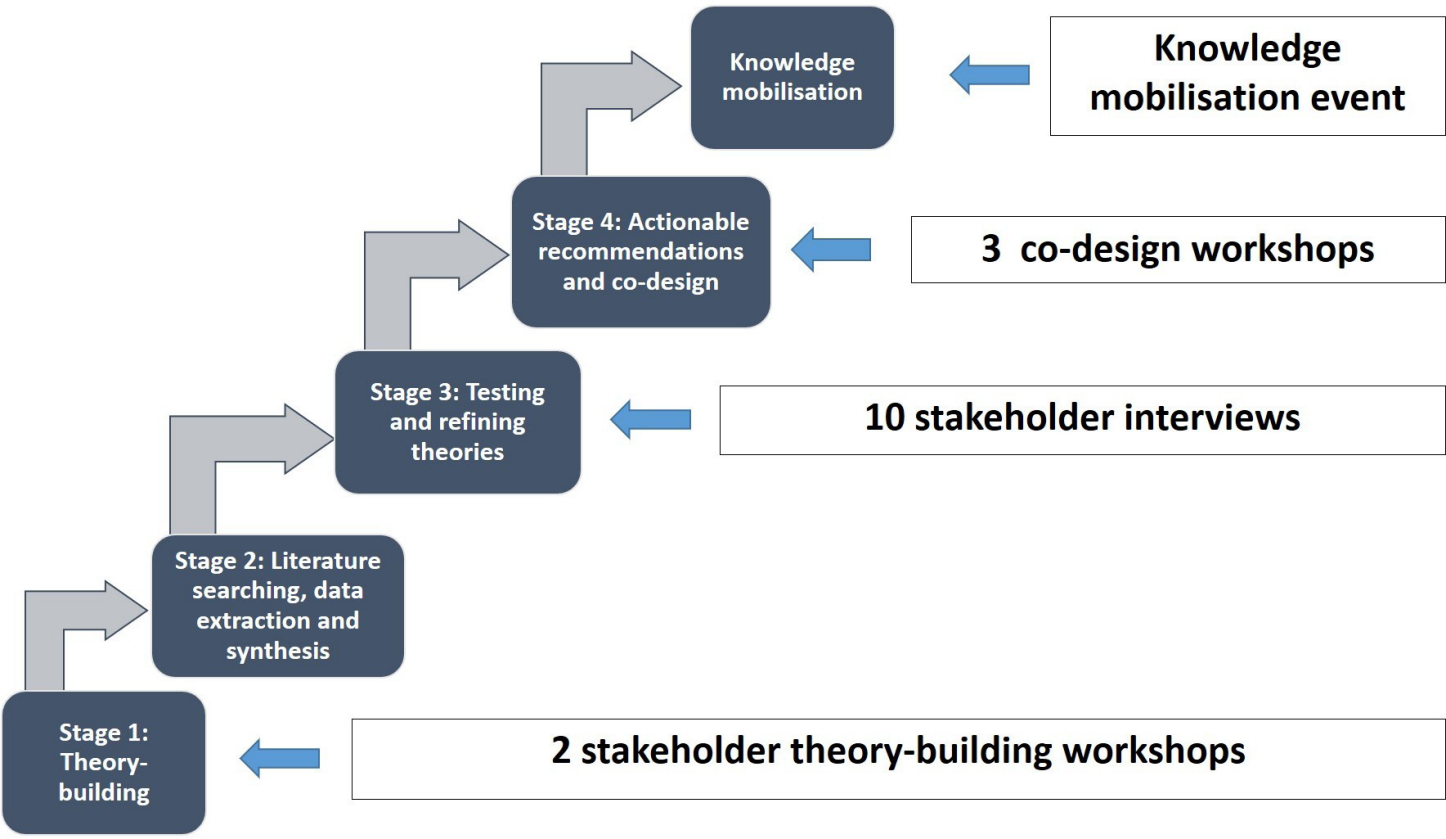
Schematic diagram of the ‘Function First’ realist evidence synthesis with embedded co-production and co-design.

This study involves four stages, detailed in the following sections and shown in figure 1.

### Stage 1: Development of initial programme theory

The first stage of the synthesis will develop initial programme theories about how and why primary care interventions aiming to improve physical function and physical activity among patients with long-term conditions work (or may not work), for whom and in what circumstances. These theories will be developed through two theory-building stakeholder workshops and a scoping review of published and grey literature.

A stakeholder analysis will identify and target the most relevant groups^44^ with representation from patients, primary care professionals working in general medical practices, policy makers, voluntary organisations, council-funded initiatives, social care, commissioners of services and National Health Service (NHS) organisations across the UK. Creative methods, borrowed from the field of co-design, will be employed to structure the workshops and elicit the views and experiences of all stakeholder representatives, including a facilitated session using LEGO® SERIOUS PLAY®. Following a series of skills-building activities, each individual will create and describe individual LEGO® models in response to the following questions: ‘What does physical function mean to you?’ and ‘What are your experiences of maintaining physical function?’ This will help to develop a shared understanding of the key topic areas and stimulate initial ideas and thoughts for theory development. These models will then be incorporated into a shared ‘landscape’ that begins to explore which aspects of these experiences helped or hindered the maintenance of physical function. Photographic images of the models will be captured and participant descriptions will be audio-recorded, and then transcribed for analysis, interpretation and shaping of emerging programme theories.

#### Theoretical landscape

The overarching theories and frameworks that are likely to inform the realist synthesis include: theories and models relating to physical function (eg, International Classification of Function^7^ environmental factors and individual compensation strategies)^45^; psychological theories of motivation, behaviour and behaviour change relevant to patients and health professionals (eg, self-efficacy and self-determination theory,^46 47^ intention and behaviour,^48^ health beliefs, planned behaviour^49 50^); interventions based around Capability, Opportunity, Motivation—Behaviour (COM-B) principles^51 52^; the self-regulation of illness^53 54^; sociological theory (eg, governmentality,^55^ habitus,^56^ social and peer support^57 58^); implementation theories (eg, diffusion,^59^ knowledge to action^60^) and organisational theories relevant to how interventions fit into different ways of delivering services and pathways.^61 62^

### Stage 2: Literature searching, data extraction and synthesis

#### Literature searching

Unlike a traditional systematic review, a realist synthesis uses a more inclusive approach involving ‘more heterogeneous evidence and an iterative process, which is less amenable to prescription but which needs to be equally rigorous’.^63^ Therefore, this stage will build on the scoping review of the literature to involve further, more purposive searches enabling the initial programme theories developed in stage 1 to be expanded.

We will review the existing literature to look for evidence to suggest how and for whom physical activity interventions work to optimise physical function in the primary care setting. It may be that interventions or services based in other areas of literature (such as secondary care, social services, the voluntary sector, or exercise science) also hold relevant insight for the development of the initial programme theories and therefore searches will not be restricted. The search strategy will be developed and amended for use with the following databases: Cochrane Library, MEDLINE, CINAHL, PsycInfo, Sociological Abstracts, Web of Science, Applied Social Sciences Index and Abstracts, Social Care Online and Social Care Institute for Excellence (see online supplementary file 1). We will also extend our searches to explore NHS reablement services (national and local) by searching the grey literature. Keywords will be developed from previous systematic reviews and the key themes, which underpin the initial programme theories, will be adapted for each information source as necessary.

10.1136/bmjopen-2019-035686.supp1 Supplementary data

We will identify references from previous relevant reviews, with forward citation tracking for key research studies. We will draw on the expertise of the project team, external project advisory group, patient and public representatives, other key researchers (nationally and internationally) and organisations to ensure that we have not missed evidence that may be relevant but not visible through traditional systematic searching methods. We will also explore the literature using cluster search methods.^64^ Where necessary, we will seek further information and clarification by contacting authors of relevant reports and relevant organisations.

Our searches will include adults of all ages and socioeconomic backgrounds. We will translate non-English language papers where relevant and practical. We will not limit our searches by publication date and there will be no restriction on the type of publication or study type that can be included. We will examine published and unpublished literature including research articles, systematic reviews and documents detailing policy and both local and national initiatives. Literature will be screened for relevance to the initial programme theories and cross-checked by two members of the research team.

We will not *s*earch for, nor include, studies that have limited transferability to NHS primary care, such as interventions involving pharmacological agents or very technical, high-cost equipment.

#### Data extraction

Consistent with the realist synthesis approach,^37^ the test for inclusion will be whether the evidence is ‘good and relevant enough’ to be included.^65^ Relevance is defined as the ability of the data to contribute to the programme theory.^66^ Assessment of relevance will involve seeking any ‘trustworthy nuggets of information to contribute to the overall synthesis’.^38^ Rigour or whether the quality of the evidence is ‘good enough’ is the research team’s judgement of the credibility of the data, including fidelity, trustworthiness and value.^67^ Bespoke data extraction forms will be designed to ensure that we capture data that informs the developing programme theories, including intervention details and any difference in implementation. If any discrepancies arise, we will discuss among the project team whether the evidence provided meets the criteria to be included.

#### Synthesis

This analytical stage will involve synthesising the evidence to elicit relationships between the contexts, mechanisms and outcomes. Based on the previous experience of the research team (CB and LW),^68–70^ suggestions from Pawson^38^ and underpinned by the principles of realist enquiry, we will use the following approach:

1. Organisation of extracted information into evidence tables representing the different bodies of literature.
2. Developing themes across evidence tables in relation to emerging patterns among the developing programme theories to seek confirming or refuting evidence.
3. Linking patterns to develop hypotheses that support or refute the developing programme theories.

Following this process, a set of synthesised statements will be formed and a narrative summarising the nature of the links between the context, mechanism and outcome will be developed (ie, what works, for whom and in what circumstances). This will also summarise the characteristics of the evidence underpinning them. This process will involve ongoing, iterative discussion among the project team members and the project advisory group.

### Stage 3: Testing and refining programme theories

In order to refine the final programme theories, we will consult with stakeholders through up to 10 telephone interviews. Purposive sampling of the stakeholders will be informed by stakeholder analysis and will aim to provide a range of perspectives from patients, primary care professionals, service delivery managers, policy makers, community-based professionals (eg, the National Exercise Referral Scheme) and commissioners. This will also enable us to capture different implementation approaches and provider influences. A semistructured interview topic guide will be used to elicit the views of stakeholders on their resonance with the developing programme theories. The approach used in the interviews will be a ‘teacher–learner cycle’ whereby the researcher presents the developing programme theories to the stakeholder (‘teaching’) and then verifies with the stakeholder where they need adjusting (‘learning’) to create an improved, refined version and a ‘mutual understanding’ of the developed programme theories.^40^ With permission, the telephone interviews will be audio-recorded and transcribed verbatim for descriptive analysis of the key themes arising during refinement of the programme theories.

The tested and refined programme theories from the evidence synthesis and stakeholder consultation will represent ‘what works’ to improve physical activity (eg, changes related to empowerment), ‘for whom’ (eg, people with long-term conditions or primary care professionals) and ‘in what circumstances’ (eg, when there are unpredictable changes in long-term condition, or limited consultation time).

### Stage 4: Intervention co-design, actionable recommendations and knowledge mobilisation

The refined programme theories will form the basis of recommendations for an intervention which is specifically designed to bring about improved physical functioning and physical activity for people with long-term conditions managed in primary care. The recommendations for service innovation, and plans for making the intervention usable, will then be designed collaboratively with stakeholders.

A team of design researchers will facilitate three consecutive co-design workshops, involving purposively sampled stakeholders including: people with long-term conditions; primary care clinicians such as general practitioners, nurses and therapists; practice managers, service delivery managers and commissioners. The three co-design workshops will ideally involve the same (or similar) people in each so that ongoing ideas can be developed and expanded during each workshop. There will be key ‘deliverables’ from each workshop, and in between workshops, designers will work to develop ideas and provocations for the next workshop, termed ‘design activities’.

#### Workshop 1 (immersion)

In this workshop, participants will immerse themselves in the lived experience of people with long-term conditions and the professional experience of people involved in primary care service and delivery. Programme theories that have been developed in the earlier stages of the review will be presented to participants. All participants will make models or images that express and visualise their own personal knowledge and experience, and how these relate to the emerging programme theories, so that they can be shared and understood by the other participants. The context will be varied for these participants, and so this workshop will also provide an opportunity for sense-checking and further refinement of the programme theories. Giving everyone the same time and space to do this at the start of a co-design process, respects and values their history and personal narrative, enabling everyone to move forward onto the main purpose of the co-design process.

*Deliverable:* A collection of models and images that represent a shared understanding and appreciation of the evidence, experiences, practice and context relevant to primary care, physical function and physical activity for people with long-term conditions.

*Design activity 1:* Between workshops 1 and 2, the designers will explore a breadth of existing interventions and analogous practices to be brought to workshop 2 as provocations for new ideas. We will also invite participants to bring examples of existing interventions or resources relating to existing interventions in which they have experience or knowledge.

#### Workshop 2 (ideation)

This will begin with a series of creative activities designed to set the tone of the workshop and simultaneously give people confidence and familiarity in these types of activities. Participants will take part in activities designed to generate and prioritise ideas and concepts using two-dimensional visualisations and sketches. These activities will use the collection of models and images developed in workshop 1, together with any provocations supplied by the designers, to generate ideas and rough prototypes of what might work. Different combinations of models and prototypes will be explored, including how they might achieve some of the ideas, or get close to achieving some of the ideas, and consequently fulfil the recommendations included in the programme theories.

*Deliverables*:

1. Generation of at least 10 concepts (eg, managing changes in long-term conditions), prioritised by workshop participants. The prioritisation will be based on immediate expert opinion (from the workshop participants together with the research project team) using simple categories of ‘novelty’, ‘technological feasibility’ (performance and manufacturing), ‘user desirability’ (ease of use, acceptability for patients and healthcare professionals) and ‘economic viability’.^48^
2. Generation of images, models or rough prototypes which could be images, sketches or three-dimensional models made out of paper, card, LEGO® or plasticine or a digital model represented through a simple animation.

*Design activity 2:* Between workshops 2 and 3, the designers will take the models or rough prototypes and make adjustments and refinements.

#### Workshop 3 (co-design)

In this workshop, the prototypes will be refined and selected. This will involve all participants testing and refining the ideas and models further and employing a shared prioritisation process to select the top three ideas. This will involve a ‘Dragon’s Den’ style activity, where participants are split into teams. Each team would further develop a concept to present back to an invited panel of ‘dragons’ (user experts) who have not been involved to date. This process provides useful critical feedback and will also be made into a ‘celebratory event’ to give participants a sense of closure.

*Deliverable:* Refinement and testing of the top three ideas for a functional intervention for primary care with one chosen following critical user feedback.

*Design activity 3:* The design team will make further adjustments based on feedback and developments from the co-design workshop.

#### Knowledge mobilisation

As this review will explore what works, for whom and in what circumstances, it is likely that the developed intervention will have core components and an ‘adaptable periphery’ that can adjust to contextual factors. A knowledge mobilisation strategy will explore these implementation variations and help to ensure that the information generated and the developed intervention are desirable (usable, acceptable, accessible), feasible (technologically, and in operational terms) and viable (economic). To assist with this, we will hold a workshop specifically dedicated to ‘knowledge mobilisation’ which will explore how best to implement this prototype intervention or new way of working, in different ways, for different contexts, thus identifying any additional resources required to support the ‘adaptation to context’ features and inform intervention design.

## Discussion

This study will add new information to this research field by conducting a realist evidence synthesis of interventions designed to improve physical activity and physical functioning for people with long-term conditions managed in primary care. The development of realist programme theory and associated intervention recommendations through an iterative co-design creative process is a new innovation. This proposal aligns with the priorities in current UK policies and recommendations^71 72^ and the findings will provide new understanding regarding how best to plan, implement and sustain physical activity interventions in primary care in order to reduce functional decline for people with long-term conditions. The synthesis findings and associated co-design outputs will lead to actionable recommendations for those involved in the organisation of health services, in particular primary care and their partners, for the benefit of patients.

Our approach to this realist evidence synthesis involves embedded co-production, using a systematic and interdisciplinary approach and involving ‘sustained engagement with stakeholders, and their systems, in order to generate implementable knowledge with impact in healthcare and health’.^73^ The realist programme theories will be developed with input from stakeholders as ‘co-producers’ throughout the review process. For example, an adapted form of LEGO® SERIOUS PLAY® will be used as a way of eliciting and sharing relevant experiences and considering collectively what made these experiences ‘successful’ or ‘unsuccessful’ forms of sustaining physical activity or physical function.

Systematic and iterative searches of relevant literature, alongside stakeholder engagement throughout this synthesis, will allow us to offer explanatory theories about the role of primary care in promoting physical activity and physical function for people with long-term conditions (including consideration of the physical, psychological and social factors that influence motivation for activity, and the value attached to physical function). The co-design and knowledge mobilisation elements will use these theories to develop desirable, feasible and viable service innovations, generating additional insight, feedback and momentum for the next ‘feasibility’ phase of the research.

We anticipate that adopting the principles of co-design as part of this synthesis, and specifically the creative practises of fully engaging people in the process at multiple time points, will nurture community–academic partnerships and facilitate eventual impact and implementation. These principles include: taking a systems perspective (ie, recognising the inter-relationships between parts of a system, rather than focusing on one part), positioning research as a creative activity with human experience at the core and considering power-sharing during the co-design process.^74 75^

### Patient and public involvement

Two patient and public research partners were involved during the proposal stage of this project, and are part of the study management group, helping to develop and refine the research objectives and methods. Two further patient and public research partners are members of the independent project advisory group. A named individual within the project team (RL) provides ongoing support for their active involvement in the following research activities: writing of the protocol and ethics application, preparation of public-facing study materials, tasks involved in development and refinement of programme theories and recommendations and dissemination. Patient and public involvement will be monitored and reported using established guidance.^76 77^

### Ethics and dissemination

Ethical considerations include, but are not limited to, informed consent, participant anonymity and confidentiality, the potential for distress, participant burden, reimbursement and honoraria and the right to withdraw from the study.

We will report our study findings using established guidance.^43^ A final report for the Health Services and Delivery Research journals library and a paper for publication in an open-access journal will be written. A key output of the knowledge mobilisation event will be content for a suite of dissemination materials, with the targeting of dissemination and methods used led by the stakeholders involved.
