# Supplementary material for: ‘Function First—Be Active, Stay Independent’—promoting physical activity and physical function in people with long-term conditions by primary care: a protocol for a realist synthesis with embedded co-production and co-design
Source: BMJ Open. 2020 Feb 9;10(2):e035686. doi: 10.1136/bmjopen-2019-035686 (PMC7045082; doi:10.1136/bmjopen-2019-035686)
Supplement: Supplementary data [file bmjopen-2019-035686supp001.pdf]

'Function First - Be Active, Stay Independent' - Promoting physical activity and physical function in people with long-term conditions by primary care: A protocol for a realist synthesis with embedded co-production and co-design.

<u>MEDLINE search strategy</u>

Ovid MEDLINE(R) and In-Process & Other Non-Indexed Citations 1946 to February 05, 2019

1. exp "Activities of Daily Living"/ OR exp Physical Fitness/ OR exp Exercise/
2. (physical function* OR functional capacity OR functional fitness OR functional decline OR functional loss* OR functional deterioration OR functional limitation*OR physical inactivity OR functional impairment OR functional decline OR sedentary).mp
3. 1 or 2
4. exp Exercise Therapy/ OR exp Physical Therapy Modalities/ OR exp Program Evaluation/
5. ((intervention* OR program* OR service* OR training OR therapy OR referral OR prescription OR self-management OR self-care OR self-monitoring OR self-efficacy OR lifestyle management OR project* OR scheme* OR initiative* OR therapies OR train*) adj5 (exercise OR physiotherapy or physical activity OR physical function*)).mp
6. (therapeutic exercise OR stay active OR goal-setting OR motivational interviewing).mp
7. 4 or 5 or 6
8. (aerobic capacity OR balance OR physical activity OR flexibility OR frailty OR functional capacity OR functional decline OR functional level OR functional power OR functional status OR frailty OR mobility OR muscle strength OR physical capability OR physical fitness OR physical function* OR physical performance OR strength OR walking capacity OR physical capacity).mp
9. exp General Practice/ OR exp Family Practice/ OR exp Primary Health Care/ OR exp Intermediate Care Facilities/ OR exp Community Health Services/ OR exp Community Health Centers/ OR exp Community networks/ OR exp Community Medicine/ OR exp Ambulatory Care/
10. (primary care OR general practi* OR family practi* OR intermediate care OR family medicine OR general medical practice OR healthcare system* OR community setting* OR community group* OR community base* OR community-based OR community intervention OR community strategy* OR community program* OR centre-based OR center-based OR ambulatory OR supervised OR rehabilitation facilit* OR long-term care OR long-term facilit* OR home care OR home healthcare OR clinic*).mp
11. 9 or 10
12. 3 AND 7 AND 8 AND 11
13. Limit 12 to (human and "all adult (19 plus years)")
